# Evaluation of the fatty acid-based erythrocyte membrane lipidome in cats with food responsive enteropathy, inflammatory bowel disease and low-grade intestinal T-cell lymphoma

**DOI:** 10.1371/journal.pone.0307757

**Published:** 2024-07-29

**Authors:** Paolo Emidio Crisi, Maria Veronica Giordano, Alessia Luciani, Alessandro Gramenzi, Paraskevi Prasinou, Anna Sansone, Valentina Rinaldi, Carla Ferreri, Andrea Boari

**Affiliations:** 1 Department of Veterinary Medicine, Veterinary Teaching Hospital, University of Teramo, Teramo, Italy; 2 Institute of Organic Synthesis and Photoreactivity, Consiglio Nazionale delle Ricerche, Bologna, Italy; University of Illinois, UNITED STATES OF AMERICA

## Abstract

Feline chronic enteropathies (FCE), include food-responsive-enteropathy (FRE), inflammatory bowel disease (IBD), and low-grade intestinal T-cell lymphoma (LGITL), and are common causes of chronic gastrointestinal signs in cats. Distinguishing between different subgroups of FCE can be challenging due to the frequent overlap of anamnestic, clinical, and laboratory data. While dysregulation in lipid metabolism has been reported in humans and dogs with chronic IBD, similar changes in cats are not yet completely understood. Assessing the fatty acid (FA) profile of red blood cell (RBC) membranes offers a valuable method for evaluating the quantity and quality of structural and functional molecular components in the membranes. Therefore, this study aimed to examine the FA composition of RBC membranes in FCE in comparison to healthy cats (HC). Gas-chromatography was used to quantitatively analyze a cluster of 11 FA, and based on these results, parameters of lipid homeostasis and enzyme activity indexes were calculated. A total of 41 FCE cats (17 FRE, 15 IBD, 9 LGITL) and 43 HC were enrolled. In FCE cats, the values of docosapentaenoic acid (p = 0.0002) and docosahexaenoic acid (p = 0.0246), were significantly higher, resulting in an overall increase in ω-3 polyunsaturated fatty acids (PUFA) (p = 0.006), and that of linoleic acid (p = 0.0026) was significantly lower. Additionally, FCE cats exhibited an increased PUFA balance (p = 0.0019) and Δ6-desaturase index (p = 0.0151), along with a decreased ω-6/ω-3 ratio (p = 0.0019). No differences were observed among cats affected by FRE, IBD and LGITL. Like humans and dogs, the results of this study indicate that FCE cats also display changes in their FA lipid profile at the level of the RBC membrane. The non-invasive analysis of RBC membrane shows promise as a potential tool for gaining a better understanding of lipid imbalances in this disease.

## Introduction

Feline chronic enteropathy (FCE) is a common gastrointestinal condition in cats, defined by persistent or intermittent GI signs of at least 3 weeks of duration, associated with intestinal inflammation, in the absence of extra-intestinal causes or infectious, obstructive, or localized neoplastic intestinal diseases [1]. These disorders are commonly classified based on treatment response into food-responsive-enteropathy (FRE), inflammatory bowel diseases (IBD) or steroid-responsive enteropathies (SRE), and low-grade intestinal T-cell lymphoma (LGITL) [1–6].

While the recognition of FRE may be easier, as it presents usually a complete clinical resolution in response to dietary intervention, distinguishing between IBD and LGITL can be challenging due to the overlapping of anamnestic, clinical, and laboratory data between the two pathologies. Current diagnosis and differentiation require complex and more invasive procedures like histopathological examination of intestinal tissue biopsies and often additional diagnostic test such as immunohistochemistry and clonality testing. Treatment often involves the use of immunosuppressive drugs [4–6]. Research efforts are focused on exploring new and less invasive diagnostic and prognostic biomarkers, as well as identifying potential therapeutic targets to improve FCE diagnosis and management.

Digestion and turnover of lipids are essential functions of the gastrointestinal tract. Lipids contain fatty acids that play different roles as major building blocks for cell membrane phospholipids, fundamental energy sources, hormones, and signaling molecules [7]. Many studies report that lipid metabolism is impaired in humans with IBD as well as in animal models of IBD [8–12]. Some clinical data suggest that phospholipids, in addition to their role in membrane formation, have many further metabolic roles (regulation of gene expression, regulation of lipid and glucose homeostasis, steroid biosynthesis, cell proliferation, and alteration of membrane’s receptors function by altering membrane fluidity [7]. Fatty acids (FAs) need to maintain their diversity, namely the appropriate presence of saturated and unsaturated structures to keep the structural properties and functional properties of cell membrane phospholipids, as well as play different roles in several other cellular compartments [7]. Studies in human medicine have shown that FAs play an important role in various phases of the inflammatory process. These include the pathogen recognition phase, in which pathogens penetrate the epithelial barrier or bond to receptors in regulating gene expression; the mobilization phase, in which immune cells immigrate from blood to the affected tissue, and the resolution phase, in which harmful agents are eliminated by anti-inflammation mediators [13]. The balance between ω-6 and ω-3 polyunsaturated FAs (PUFAs) and the ratio of saturated and unsaturated FAs are therefore crucial in regulating signaling outcomes and membrane properties [14–16].

The FA profiles have been investigated in different biological samples, such as serum, whole blood, urine, tissues, and stools, using different techniques in human patients with ulcerative colitis (UC) and Chron Disease (CD) [8, 16–22].

Recently, a FA-based RBC membrane lipidomic analysis was conducted in dogs with chronic enteropathy (CE), which revealed significant differences in the lipidomic profile of erythrocytes between dogs with CE and healthy dogs, such as higher levels of stearic acid, dihomo-gamma-linolenic (DGLA), eicosapentaenoic acid (EPA), and docosahexaenoic acid (DHA), and lower levels of palmitic and linoleic acids in CE dogs [23].

To date, knowledge is limited about the lipidomic profile of RBC membranes in cats and the authors hypothesize that the RBC membrane lipidome may mirror the “gut health” of cats affected by chronic enteropathy, as already observed in dogs. A recent study demonstrated the presence of lipid maldigestion and malabsorption in FCE [24] and the understanding of the lipid alterations can provide insights into the FCE mechanisms with potential future therapeutic applications. For these reasons the present study aimed to investigate the differences in the quantity and quality of RBC membrane FAs between healthy cats and those with chronic gastrointestinal diseases.

## Material and methods

### Study population and diagnostic investigations

Cats admitted to the Veterinary Teaching Hospital (VTH) of the University of Teramo between January 2018 and November 2022 were prospectively enrolled in the study. The project was approved by the Committee on Animal Research and Ethics of the Universities of Chieti-Pescara, Teramo, and Experimental Zooprophylactic Institute of AeM (CEISA), Protocol UNICHD12 n. 1168. Prior to enrollment in the study, cat owners provided written informed consent.

Cats with persistent or intermittent clinical signs of chronic enteropathy lasting for at least 3 weeks, were eligible for enrollment in the group of cats with chronic enteropathy (FCE). Extra-gastrointestinal disease, infectious and parasitic intestinal diseases and localized neoplastic intestinal lesions were excluded based on a history, physical examination (including a 9-point BCS), complete blood count (CBC), serum biochemistry profile (i.e. glucose, blood urea nitrogen, creatinine, total bilirubin, aspartate aminotransferase, alanine aminotransferase, γ-glutamyl transferase, serum alkaline phosphatase, creatin kinase, DGGR lipase, calcium, phosphorus, albumin, total proteins, cholesterol, triglycerides, sodium, potassium, chloride, magnesium), urinalysis, total T4 (for cats > 6 years of age), feline serum pancreatic lipase (fPLI) and feline trypsin-like immunoreactivity (fTLI), a direct fecal smear evaluation and zinc sulfate centrifugal flotation, and abdominal ultrasound.

Exclusion criteria were antiacids or antibiotic treatment in the previous 6 months, and the presence of systemic or extra-gastrointestinal disease. Also, cats that have received dietary ω-3 supplementation in the last 4 months were not included in the present study.

In FCE cats, when available, serum cobalamin and folate concentrations, FCE activity index (FCEAI) [25] were recorded.

In the same period, examined cats without clinical or clinic-pathological evidence of disease, determined by a comprehensive assessment that included medical history, physical examination, CBC, serum biochemistry and urinalysis, were included as healthy cats (HC).

FCE cats were classified as having food-responsive enteropathy (FRE), idiopathic inflammatory bowel disease (IBD), and low-grade intestinal T-cell lymphoma (LGITL) [1–3]. In particular, the group of FRE includes patients who experienced complete remission of gastrointestinal symptoms within 3 weeks of a dietetic trial with a single novel protein source or hydrolyzed protein diet [26]. The remission was considered complete if clinical signs were resolved or the FCEAI score was reduced by ≥75% after three weeks of dietary therapy [25]. Patients who failed to respond to at least two dietary trials underwent gastro-duodenoscopy and ileo-colonoscopy for diagnostic purposes and biopsy specimens were evaluated by board-certified pathologists.

When an underlying LGITL was suspected by the pathologist based on histopathology, additional CD3 and CD20 immunohistochemical staining and clonality test [4–6] were performed to confirm the diagnosis. A final diagnosis of IBD or LGITL was reached by integrating the results from histopathology, immunohistochemistry, and clonality test. The same therapeutic diet was continued throughout the trial in all FCE cats.

Moreover, according to the folates and cobalamin levels, FCE cats were classified as hypofolatemic, normofolatemic or hyperfolatemic (reference interval 9.7–21.6 μg/L), and hypocobalaminemic, normocobalaminemic and hypercobalminemic (reference interval 290–1500 ng/L).

### Fatty acid-based erythrocyte membrane lipidome analysis

At the time of the admission, from each cat, blood samples were collected from the jugular vein using EDTA tubes.

The isolation of phospholipids from RBC membranes and transesterification procedure, gas-chromatographic analysis of fatty acid methyl esters (FAME) and calibration procedure were performed as previously described for dogs [23, 27].

Gas-chromatography has a flame- ionization detector (FID) which allows to quantify the fatty acids by having standard references for each of the fatty acid of the mixture.

A cluster of 11 FAs, representative of the main FA moieties present in the cell membrane, was chosen (Table 1). In particular, the cohort of FAs included: palmitic (C16:0) and stearic (C18:0) acids as SFAs; palmitoleic (C16:1), oleic (9c, C18:1), and vaccenic (11c, C18:1) acids as MUFAs; linoleic (LA, C18:2), DGLA (C20:3), and arachidonic (AA, C20:4) acids as ω-6 PUFA; EPA (C20:5), docosapentaenoic acid (DPA, C22:5) and DHA (C22:6) as ω-3 PUFA. These values were also reported as total FA contents (total SFA, total MUFA, total PUFA, total ω-6, total ω-3).

**Table 1:** Cluster of individual fatty acids determined in study cats and related families. Together with the common names, the abbreviations describe the position and geometry of the double bonds (e.g., 9c for palmitoleic acid), as well as the notation of the carbon chain length and total number of double bonds (e.g., C18:1).

Families of Fatty Acids		Individual Fatty Acids
Saturated Fatty Acids		Palmitic acid (C16:0)
		Stearic acid (C18:0)
Monounsaturated Fatty Acids		Palmitoleic Acid (C16:1)
		Oleic Acid (9c, C18:1)
		Vaccenic Acid (11c, C18:1)
Polyunsaturated Fatty Acids	ω-6 Polyunsaturated Fatty Acids	Linoleic Acid (C18:2)
		Dihomo-gamma-linolenic Acid (C20:3)
		Arachidonic Acid (AA, C20:4)
	ω-3 Polyunsaturated Fatty Acids	EPA (C20:5)
		DPA (C22:5)
		DHA (C22:6)

EPA: eicosapentaenoic acid; DPA: docosapentaenoic acid; DHA: docosahexaenoic acid (DHA),

Lipid indexes were calculated as follows: ω-6-to-ω-3 ratio (ω-6/ω-3), PUFA balance [ω-3/(ω-3 + ω-6)], SFA-to-MUFA ratio (SFA/MUFA), unsaturation index (UI = MUFA total x 1 + C18:2 x 2 + C20:3 x 3 + C20:4 x 4 + C20:5 x 5 + C22:6 x 6) and peroxidation index (PI = MUFA total x 0.025 + C18:2 x 1 + C20:3 x 2 + C20:4 x 4 + C20:5 x 6 + C22:6 x 8) [23, 27]. The elongase-6 activity index (EI = C18:0/C16:0), Δ-9 desaturase activity index (Δ9DI = 9c, C18:1/C18:0), Δ-6 desaturase activity index (Δ6DI = C20:3/C18:2), and Δ-5 desaturase index (Δ5DI = C20:4/C20:3) were calculated as precursors to FA ratios, as already described in dogs [23].

## Statistical analysis

The computer software GraphPad Prism version 6.01 (GraphPad Software San Diego, CA) was utilized to perform the statistical analysis. All data were evaluated using a standard descriptive statistic and reported as mean ± SD or as median and interquartile range, based on their distribution. Normality was checked using the D’Agostino Pearson test. For comparison between two groups, the unpaired t-test or Mann-Whitney test was used, while for comparison among more than two groups, ANOVA or Kruskal-Wallis test and post-hoc tests (Student-Newman-Keuls test or Dunn test) were employed. Correlations between FA percentages, homeostasis indexes or enzyme activity indexes with FECAI and BCS were evaluated by Spearman or Pearson correlation analysis depending on their distribution. The threshold of statistical significance was set at *p* < 0.05.

Principal coordinate analysis and hierarchical clustering heatmaps were generated using R Studio and Metaboanalyst 5.0, respectively.

## Results

### Study population

Eighty-three cats, of which 41 were diagnosed with FCE and 43 were HC, underwent FA-based RBC membrane lipidome analysis (Tables 2 and 3).

**Table 2:** Breed, sex, age, body weight and diagnosis of the recruited cats with feline chronic enteropathy (n = 41).

	Breed	Sex	Age (month)	Body weight	Diagnosis
1	Domestic Shorthair	Mn	20	4.6	IBD
2	Domestic Shorthair	Fs	144	3	FRE
3	Domestic Shorthair	Fs	132	3	IBD
4	Domestic Shorthair	Fs	108	3	LGITL
5	Domestic Shorthair	Mn	48	4	IBD
6	Domestic Shorthair	M	34	3.6	FRE
7	Domestic Shorthair	Fs	60	2.7	FRE
8	Domestic Shorthair	F	120	3	LGITL
9	Domestic Shorthair	Mn	120	7.3	FRE
10	Domestic Shorthair	Mn	18	5.2	FRE
11	Siamese	Fs	48	3.9	FRE
12	Domestic Shorthair	Fs	8	2	FRE
13	Domestic Shorthair	Mn	108	3.65	LGITL
14	Domestic Shorthair	Mn	18	2.4	FRE
15	Domestic Shorthair	Mn	84	6.9	FRE
16	Domestic Shorthair	Fs	24	2.25	FRE
17	Domestic Shorthair	Fs	120	4.9	FRE
18	Domestic Shorthair	Fs	120	6.7	FRE
19	Domestic Shorthair	Mn	120	3.6	LGITL
20	Domestic Shorthair	M	156	4	LGITL
21	Domestic Shorthair	Mn	147	3.3	LGITL
22	Domestic Shorthair	Fs	182	4.1	LGITL
23	Domestic Shorthair	Fs	108	3.3	FRE
24	Domestic Shorthair	Fs	48	3	IBD
25	Domestic Shorthair	Fs	96	6.3	IBD
26	Domestic Shorthair	Mn	155	3	LGITL
27	Domestic Shorthair	M	138	5.4	IBD
28	Domestic Shorthair	Mn	128	5.3	IBD
29	British Shorthair	Mn	36	3.9	IBD
30	Maine Coon	Mn	60	5	IBD
31	Abyssinian	Mn	117	4.1	FRE
32	Domestic Shorthair	Fs	96	4.1	IBD
33	Domestic Shorthair	Mn	124	3	IBD
34	Domestic Shorthair	Fs	120	3.2	IBD
35	Carthusian	Fs	120	3.2	IBD
36	Domestic Shorthair	Mn	120	7.2	FRE
37	Domestic Shorthair	Fs	156	3.4	LGITL
38	Domestic Shorthair	Mn	58	4.3	FRE
39	Main coon	F	132	4.2	IBD
40	Domestic Shorthair	M	5	7	FRE
41	Devon rex	M	68	2	IBD

Male; F: Female; Mn: Neutered male; Fs: Spayed female; FRE: Food-responsive enteropathy; IBD: inflammatory bowel disease; LGITL: low-grade intestinal T-cell lymphoma.

**Table 3:** Breed, sex, age, body weight and diagnosis of the recruited healthy cats (n = 43).

	Breed	Sex	Age (month)	Body weight
1	Domestic Shorthair	Fs	55	4
2	Domestic Shorthair	Fs	69	5
3	Domestic Shorthair	Fs	136	2.5
4	Domestic Shorthair	F	19	3.6
5	Domestic Shorthair	Fs	83	3.3
6	Domestic Shorthair	Fs	9	3
7	Domestic Shorthair	Mn	36	4.2
8	Siamese	Mn	23	4.5
9	Domestic Shorthair	Fs	132	5.7
10	Domestic Shorthair	M	60	5
11	Domestic Shorthair	Fs	60	3
12	Domestic Shorthair	Fs	10	3.5
13	Domestic Shorthair	Mn	36	4.5
14	Domestic Shorthair	Mn	65	5.8
15	Domestic Shorthair	Fs	7	3.2
16	Domestic Shorthair	Mn	5	4.7
17	Domestic Shorthair	Fs	72	3
18	Domestic Shorthair	Mn	24	4
19	Domestic Shorthair	Fs	24	4.5
20	Domestic Shorthair	M	36	4.25
21	Domestic Shorthair	Fs	24	2.3
22	Domestic Shorthair	Mn	24	3.8
23	Domestic Shorthair	F	12	2.1
24	Domestic Shorthair	F	24	2.2
25	Domestic Shorthair	Mn	12	3.3
26	Domestic Shorthair	F	12	1.8
27	Domestic Shorthair	F	37	3
28	Domestic Shorthair	F	13	2.4
29	Domestic Shorthair	Fs	33	2.3
30	Domestic Shorthair	F	45	2.5
31	Domestic Shorthair	M	57	4.2
32	Domestic Shorthair	M	33	2.8
33	Domestic Shorthair	Mn	36	4.2
34	Domestic Shorthair	Mn	60	5
35	Domestic Shorthair	Fs	120	3.4
36	Domestic Shorthair	Mn	132	3.8
37	Domestic Shorthair	Mn	108	4.1
38	Domestic Shorthair	Fs	163	3.8
39	Domestic Shorthair	Mn	163	5.2
40	Domestic Shorthair	Fs	118	4.8
41	Domestic Shorthair	Mn	67	6.1
42	Domestic Shorthair	M	9	4.2
43	Domestic Shorthair	Mn	19	4.7

M: Male; F: Female; Mn: Neutered male; Fs: Spayed female

Among the FCE group, 17 cats responded positively to the dietary trial and were diagnosed with FRE. Of the 24 cats that failed to respond positively to at least two dietary trials, 15 were diagnosed with IBD, and 9 with LGITL, based on biopsy and histopathological evaluations.

Among the FCE cats, the FCEAI was available in 34, with a mean value of 7.6 ± 3.9. Folates and cobalamin levels were available in 31 cats; 14 had cobalamin below the RI (290–1500 ng/L), 17 had cobalamin levels within the RI, while no cats were hypercobalaminemic 9 had folate below the RI, 16 had folate levels within the reference interval, and 6 had folate levels above the reference interval. The clinical findings observed in FCE are summarized in Table 4.

**Table 4:** Clinical and clinicopathological findings observed in cats with chronic enteropathy.

Variables	n (%)
Vomiting	22/41 (53.6%)
Diarrhea	22/41 (53.6%)
Weight loss	22/41 (53.6%)
Decreased serum cobalamin (< 290 ng/l)	14/31 (45.1%)
Decreased serum folate (< 9.7 μg/l)	9/31 (29%)
Decreased appetite	10/41 (24.3%)
Increased serum folate (> 21.6 μg/l)	6/31 (19.3%)
Decreased attitude/activity	2/41 (4.88%)

### Fatty acid-based erythrocyte membrane lipidome analysis

**Fig 1:**
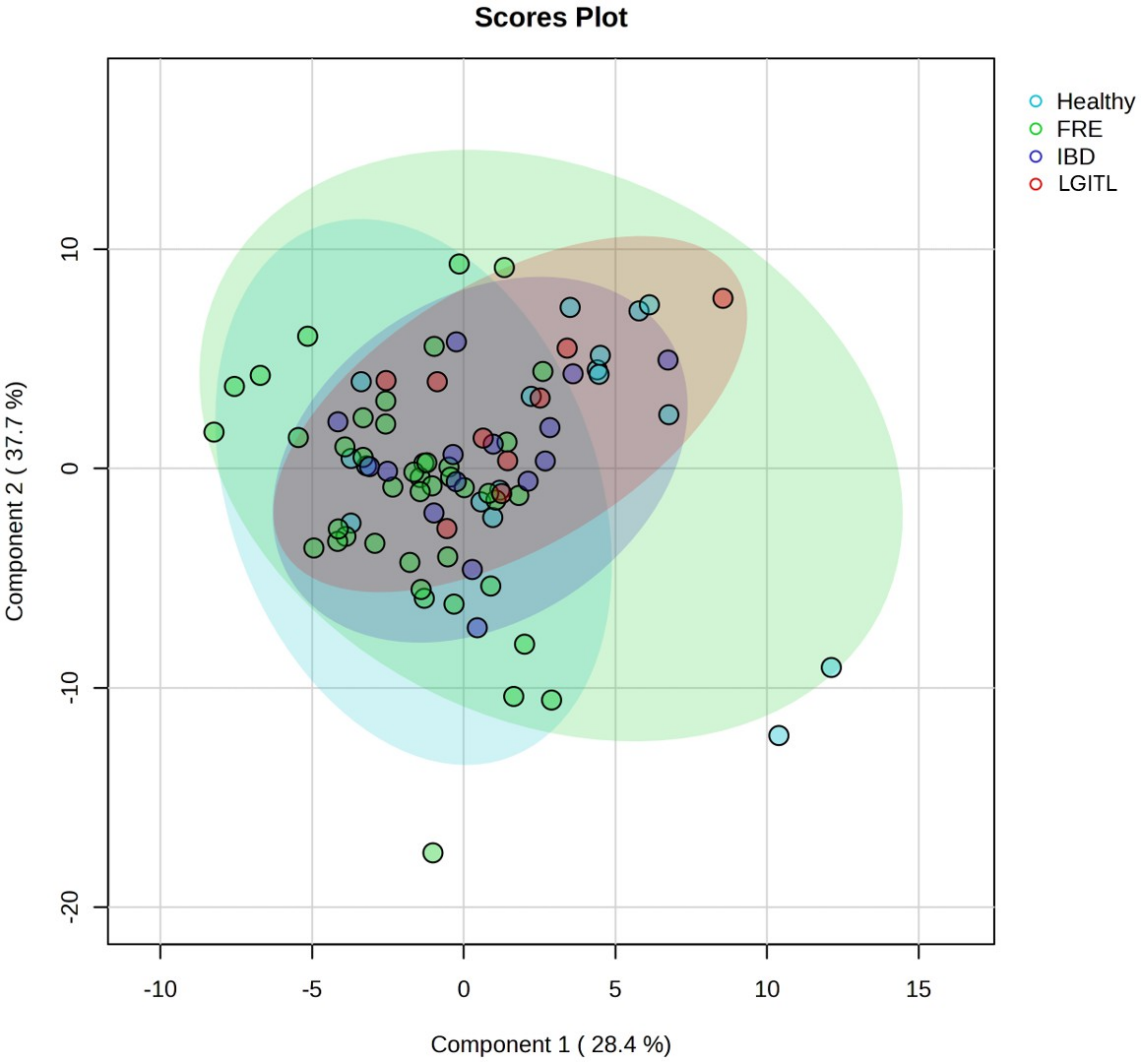
Plots of the scores for the two principal components (variance explained: 28.4% and 37.7%, respectively) of healthy cats and cats with food-responsive enteropathy (FRE), inflammatory bowel disease (IBD) and low-grade intestinal T-cell lymphoma (LGITL).

**Fig 2:**
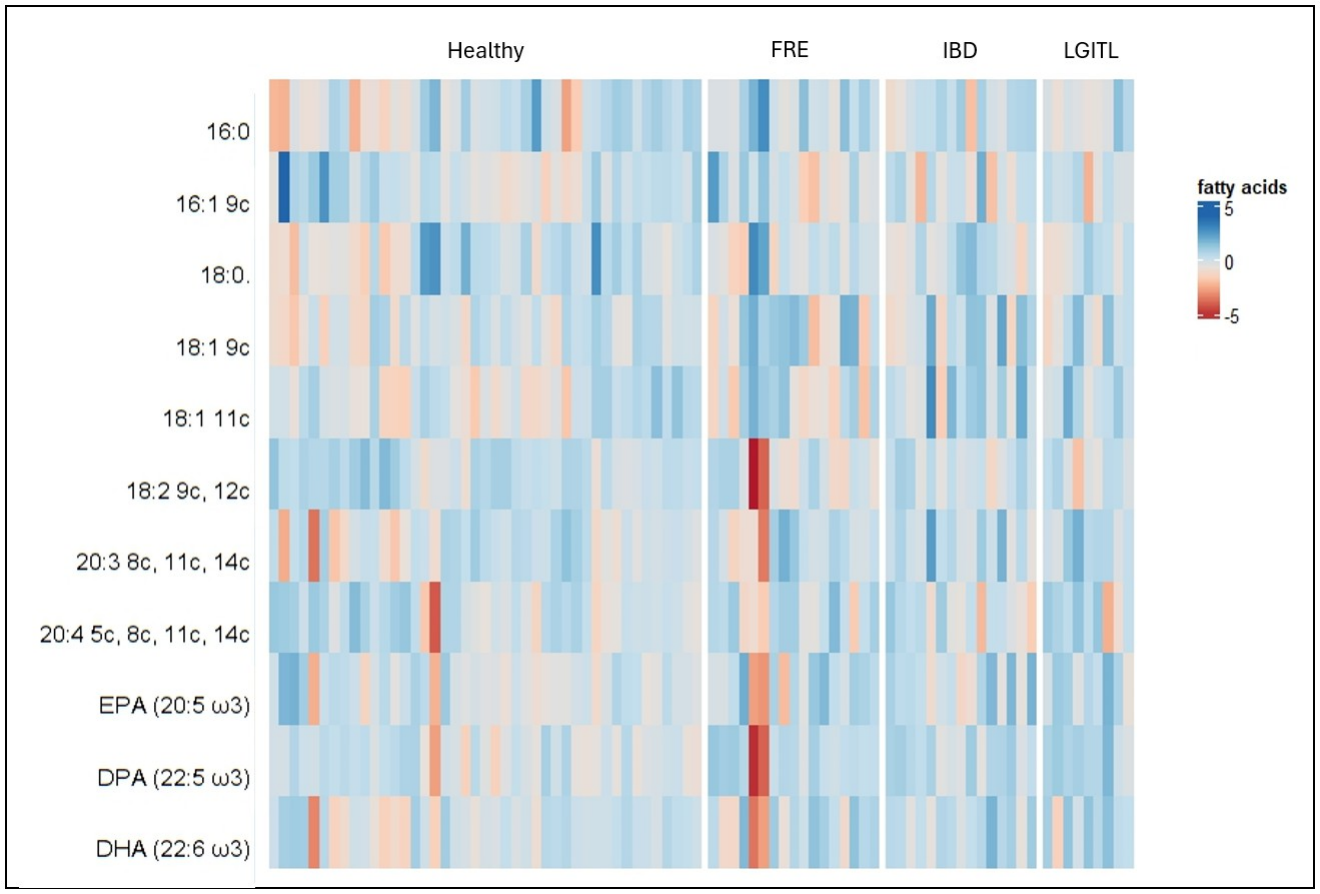
Heatmap of the fatty acid of red blood cells membranes. Each row represents the intensity of one fatty acid; each column represents one sample. The higher the concentration of a fatty acid, the more intensely red shows. The lower the signal intensity of a fatty acid, the more intensely blue the metabolite shows in the heatmap.

Median values with interquartile ranges of the single FAs, total FA contents (total SFAs, total MUFA, and total PUFA), homeostasis indexes (SFA/MUFA, ω-6/ω-3, UI, PI, and PUFA balance) and enzyme activity indexes (EI, Δ9DI, Δ6DI, Δ5DI) of FCE cats are reported in Table 5. Graphical representations of variations in fatty acids among the three groups of cats, is presented as principal coordinate analysis (Fig 1) and hierarchical clustering heatmaps (Fig 2).

**Table 5:** Median values with interquartile ranges in brackets of the single FAs, total FA contents of red blood cells membranes (total SFA, total MUFA, and total PUFA), homeostasis indexes (SFA/MUFA, ω-6/ω-3, UI, PI, and PUFA balance) and enzyme activity indexes (EI, Δ9DI, Δ6DI, Δ5DI) for both healthy cats and cats with FCE. Statistically significant *p*-values (*p* < 0.05) are highlighted in bold.

Variable	Healthy Cats (n = 43) Median value (IQR)	FCE (n = 41) Median value (IQR)	p value
C16:0—Palmitic Acid	18.97 (16.1–22.3)	19.1(17.4–22.6)	0.4760
C16:1—Palmitoleic Acid	0.17 (0.11–0.22)	0.14 (0.10–0.20)	0.3234
C18:0—Stearic Acid	20.4 (23–24.6)	22.4 (20.7–24.3)	0.6498
9c,C18:1—Oleic Acid	8.32 (9.23–10)	9.94 (0.05–12.1)	0.0892
11c,C18:1—Vaccenic Acid	1.45 (1.73–1.96)	1.82 (1.50–2.27)	0.1204
LA ω-6—C18:2—Linoleic Acid	21.0 (23.2–25.4)	20.6 (17.8–23.5)	**0.0026**
DGLA ω-6—C20:3 Dihomogammalinolenic Acid	0.54 (0.75–0.92)	0.80 (0.64–1.06)	0.2042
ARA ω-6—C20:4—Arachidonic Acid	19.3 (16.1–23.3)	19.9 (14.8–23.7)	0.9840
EPA ω-3—C20:5—Eicosapentaenoic Acid	0.90 (0.64–1.50)	1.44 (0.66–2.58)	0.0555
DPA ω-3—C22:5—Docosapentaenoic Acid	0.50 (0.34–0.67)	0.74 (0.51–0.74)	**0.0002**
DHA ω-3—C22:6—Docosahexaenoic Acid	0.90 (0.69-1-31)	1.36 (0.62–1.98)	**0.0246**
Total SFA	42.5 (36.5–46.2)	41.4 (39.8–45.4)	0.9947
Total MUFA	11.3 (10.28–11.9)	11.9 (9.67–14.5)	0.0939
Total PUFA	46.55 (41.5–51.5)	45.5 (42.1–49.3)	0.4942
ω-6 PUFA	43.6 (39.0–48.5)	41.1 (36.7–45)	0.0552
ω-3 PUFA	2.35 (1.87–2.87)	3.48 (2.29–5.36)	**0.0026**
ω-6/ω-3 ratio	19.7 (14.6–22.1)	13.0 (7.82–19.2)	**0.0019**
SFA/MUFA	3.79 (3.43–4.15)	3.64 (2.85–4.21)	0.2687
PUFA balance	4.82 (4.32–6.38)	7.12 (4.93–11.35)	**0.0019**
UI	151.9 (136.1–167.5)	158.6 (139.8–170)	0.4651
PI	122.2 (105.4–139.1)	131.8 (110.4–147.1)	0.1055
Elongase-6 activity	1.22 (0.58–1.37)	1.16 (1.01–1.28)	0.1428
Delta-9 desaturase	0.40 (0.35–0.43)	0.42 (0.34–0.52)	0.1135
Delta-6 desaturase	0.32 (0.02–0.04)	0.03 (0.02–0.060)	0.0151
Delta-5 desaturase	23.9 (20.1–32.6)	23.13 (17.1–30.9)	0.4022

IQR: interquartile range; SFA: Saturated Fatty Acids; MUFA: Monounsaturated Fatty Acids; PUFA: Polyunsaturated Fatty Acids; UI: Unsaturation index; PI: Peroxidation index.

**Fig 3:**
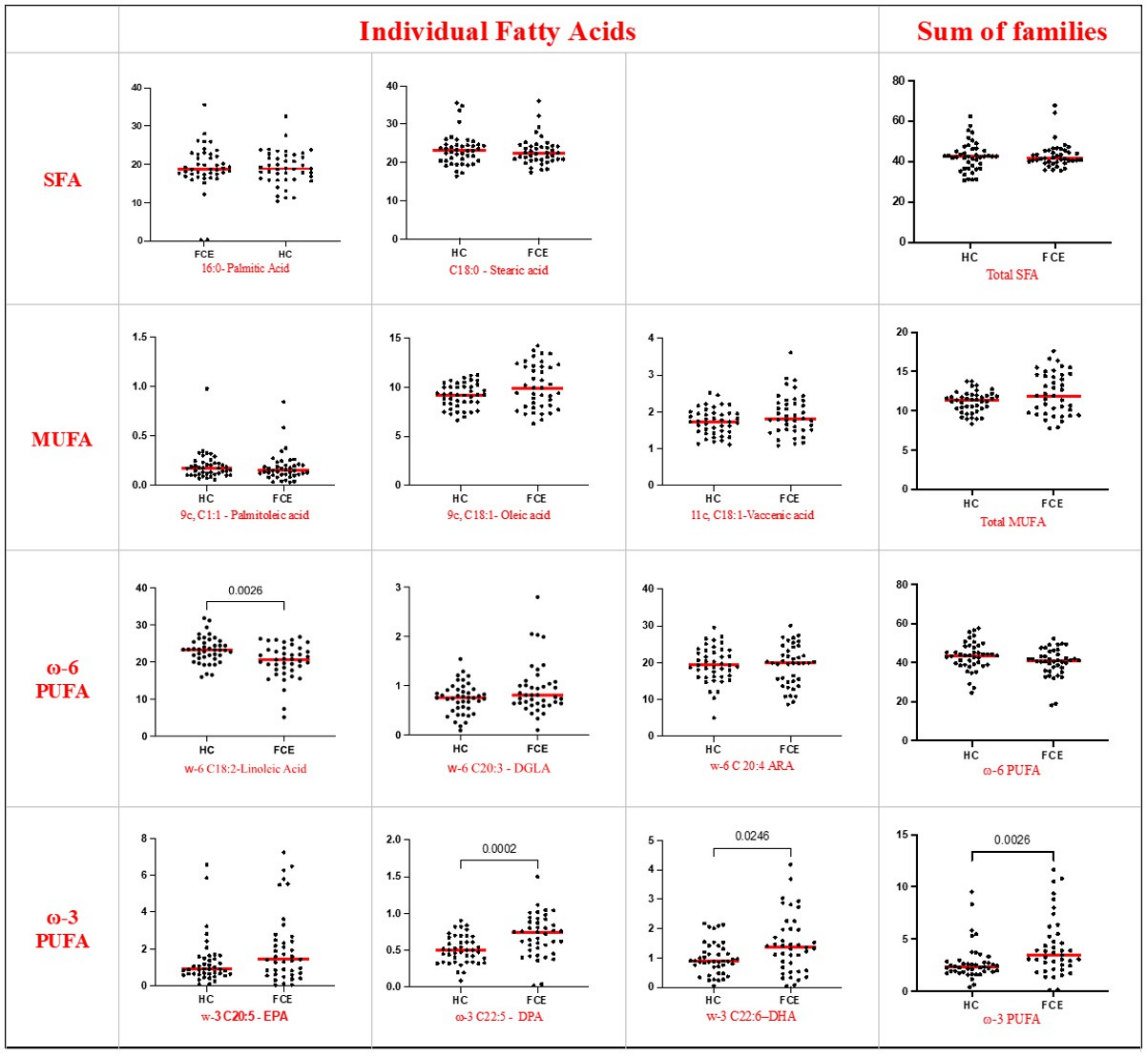
Red blood cell membranes concentration of fatty acids in healthy cats (n = 43) and cats with chronic enteropathy (n = 41). Red lines represent the median. Statistically significant p-values (p-value <0.05) are showed in thew graphs. SFA: Saturated Fatty Acids; MUFA: Monounsaturated Fatty Acids; PUFA: Polyunsaturated Fatty Acids.

**Fig 4:**
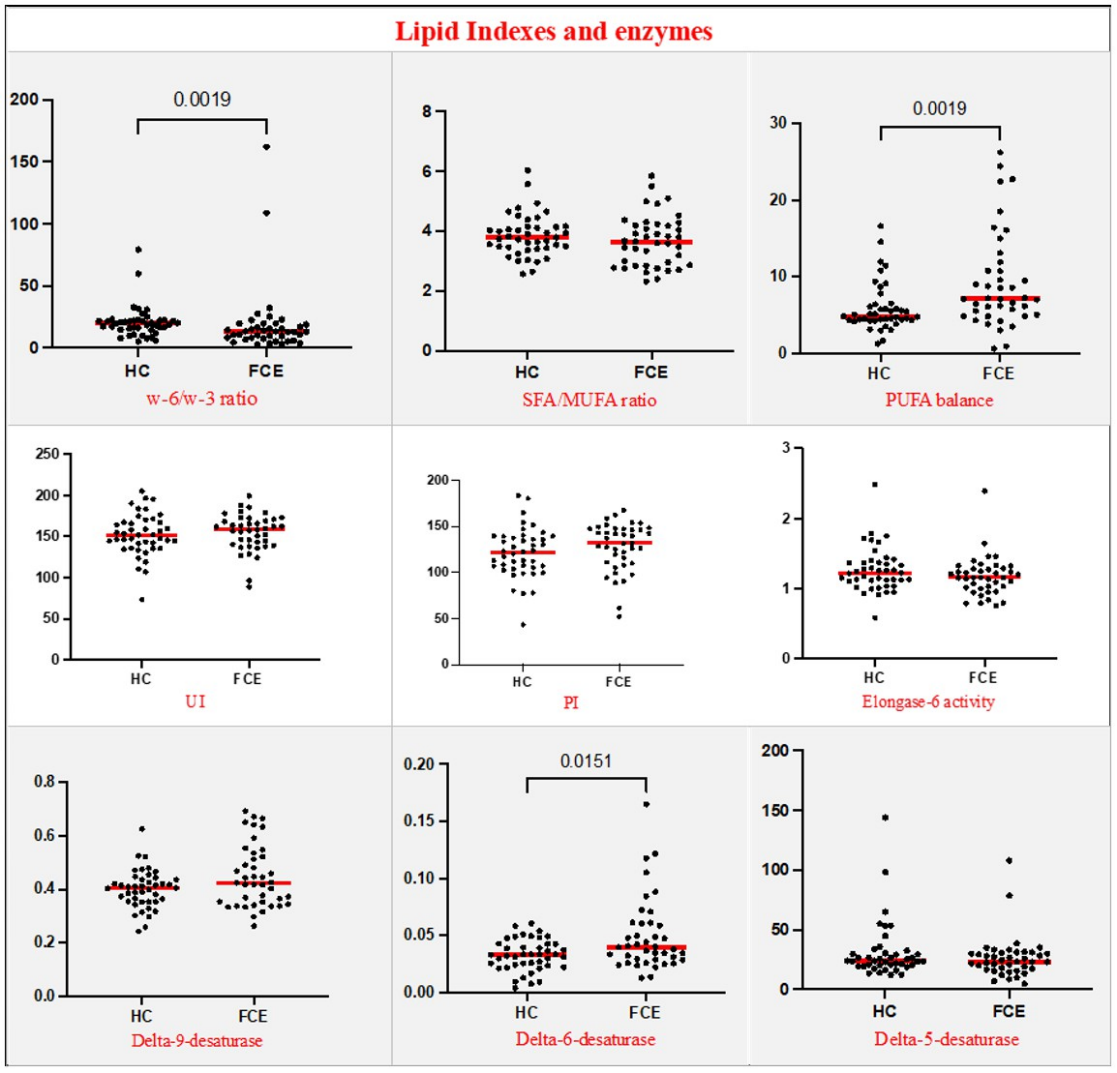
Fatty acids indexes and enzymes indexes in healthy cats (n = 43) and cats with chronic enteropathy (n = 41). Red lines represent the median. Statistically significant p-values (p-value <0.05) are showed in the graphs. SFA: Saturated Fatty Acids; MUFA: Monounsaturated Fatty Acids; PUFA: Polyunsaturated Fatty Acids; UI: Unsaturation index; PI: Peroxidation index.

Compared to HC, in the RBC membranes of FCE cats higher values of DPA (p = 0.0002) and DHA (p = 0.0246) were observed, with an overall increase of RBC content of ω-3 PUFA (p = 0.006), and lower values of RBC content of ω-6 PUFA linoleic acid (p = 0.0026). Additionally, the membrane homeostasis index patterns in FCE cats showed an increased PUFA balance (p = 0.0019) and a decreased ω-6/ω-3 ratio (p = 0.0019); also, Δ6DI resulted increased in FCE cats (p = 0.0151) (Figs 3 and 4).

Regarding the diagnosis, no differences were observed in FA-based RBC membrane lipidome among FRE, IBD, and LGITL cats (S1 Table).

In comparison with FCE cats with normal levels of serum cobalamin (n = 17), hypocobalaminemic FCE cats (n = 14) had decreased values of total PUFA (p = 0.0467), SFA/MUFA ratio (p = 0.0005), and the value of PI (p = 0.0467), while the values of oleic acid (p = 0.0030) and vaccenic acid (p = 0.0155), total MUFA (p = 0.0013), Δ9DI (p = 0.0008) and Δ5DI (p = 0.003) were increased. No differences in RBC membrane fatty acids levels, lipid indexes and enzyme indexes were observed among hypofolatemic, normofolatemic, and hyperfolatemic cats.

Linoleic acid (r = 0.43, 95% CI [0.11; 0.67], n = 34, p = 0.01), DPA (r = 0.47, 95% CI [0.16; 0.42], n = 34, p = 0.004), ω-6 PUFA (r = 0.38, 95% CI [0.04; 0.63], n = 34, p = 0.03) and total PUFA (r = 0.42, 95% CI [0.09; 0.66], n = 34, p = 0.01) were positively correlated with the FCEAI, while a negative correlation with FCEAI was observed for palmitic (r = -0.40, 95% CI [-0.65; -0.07], n = 34, p = 0.01), oleic acids (r = -0.30, 95% CI [-0.63; -0.04], n = 34, p = 0.02) and SFA (r = -0.35, 95% CI [-0.61; -0.01], n = 34, p = 0.04).

The BCS was found positively correlated with DHA (r = 0.33, 95% CI [0.01; 0.58], n = 41, p = 0.03), total ω-3 (r = -0.31, 95% CI [0.01; 0.56], n = 41, p = 0.04) and PUFA balance (r = 0.32, 95% CI [0.01; 0.57], n = 41, p = 0.04), while a negative correlation with ω-6/ω-3 (r = -0.32, 95% CI [-0.57; -0.001], n = 41, p = 0.04) (p = 0.0442, r = − 0.32) was observed.

## Discussion

The results of the present study revealed distinct differences in the FA composition of RBC membranes between FCE cats and the HC group. The most consistent differences observed in FCE compared to healthy ones were related to PUFA metabolism. In FCE, increased DPA and DHA levels and, consequently, total ω-3 PUFA, were observed, alongside a decrease in LA levels. Accordingly, increased PUFA balance and Δ6DI and reduced ω-6/ω-3 ratio was also recorded. These results are consistent with those observed in dogs with CE [23], supporting the hypothesis that the lipidic imbalance observed in CE is very similar in the two species, despite clear metabolic differences. Indeed, cats possess limited Δ6 desaturase activity and alternative enzymatic pathways mediated by Δ-5 and Δ-8 desaturase to produce AA have been evocated [28]. For this reason, cats have a greater requirement for dietary AA, EPA, and DHA, than dogs, especially for pregnant and lactating cats for offspring’s brain and nervous system development. The FEDIAF Nutritional Guidelines for Complete and Complementary Pet Food for Cats and Dogs recommend the following FAs levels for complete cat food Unit per 1000 kcal of metabolizable energy (ME), based on ME Requirements of 75 Kcal/Kg^0.67^: 1.67 grams of LA and 20 mg of AA for adult cats, and 1.38 grams of LA, 50 mg of AA, 0.05 grams of α-linolenic acid and 0.03 grams of EPA+DHA [29]. In contrast, dogs, with their active Δ6 desaturase, efficiently convert linoleic acid to arachidonic acid, and can synthesize EPA and DHA from α-linolenic acid. Additionally, dogs exhibit high stearoyl-CoA desaturase activity, aiding in the conversion of palmitic acid to unsaturated fatty acids [28].

Regarding moisture content, there is a lack of available data concerning the bioavailability of FAs in cats consuming heat-treated canned food. However, recent research indicates that moisture levels may influence the production of short-chain FAs, potentially attributable to augmented substrate reaching the colon for fermentation, particularly observed in cats consuming a dry diet [30].

Interestingly, as already observed in dogs [23], the total ω-6 and AA levels did not differ in the RBC membranes of FCE and HC, further recalling the complex biochemical relationship between ω-3 and ω-6 pathways in pathological conditions. Indeed, the balance between ω-6 and ω-3 PUFA is critical for regulating the signaling outcome in chronic enteropathy [31].

While few studies in cats evaluated the effects of diet on different blood compartments (i.e. serum, plasma, RBC membrane) and tissue [32, 33], to the authors’ knowledge, this is the first study investigating the FA cohort of the RBC membrane lipidome in cats affected by CE. A cluster of 11 FAs representative of each family, including SFA, MUFA, and PUFA, was selected based on their structural and functional roles, and their relative abundance in the RBC membrane, since it belongs to the most representative FAs of the membranes, accounting for approximately 97% of the total content.

The analysis of FA levels in plasma or serum has been extensively conducted as it provides insights into short-term dietary fat consumption [34]. Nonetheless, examining the lipid composition in RBC membranes offers distinct advantages over plasma analysis. This is primarily because erythrocytes have a longer lifespan (i.e. approximatively 120 days in humans and dogs, 65–75 days in cats) in the bloodstream making them a more reliable indicator of long-term dietary FA intake and overall tissue conditions [34]. Additionally, it’s worth noting that erythrocytes tend to maintain a more consistent and stable FA composition compared to the levels found in plasma, as it is generally believed that these cells keep their FA distribution throughout their life. [35].

Moreover, FA-based RBC membrane lipidome may provide an estimation of the clinical and nutritional status of the patients, and of the metabolic pathways in which they are mostly involved [36]. In particular, the SFA and MUFA levels provide information on the liponeogenesis and Δ-9 desaturase activity, while essential PUFA are indicative of the nutritional status, and their metabolites indicate the efficiency of further steps of elongation, Δ-6 and Δ-5 desaturations [37–40].

The result of the present study suggested a dysregulation in the balance between ω-3 and ω-6 in cats with CE. The role of FAs in inflammation is central to the understanding of the pathophysiology of various conditions, including chronic inflammatory gastrointestinal disorders. In this context ω-6 PUFA, specifically LA, are recognized as key precursors for the biosynthesis of AA and a multitude of inflammatory mediators, including prostaglandins and leukotrienes, which are well-documented for their pro-inflammatory properties [41]. On the contrary, ω-3 PUFA are acknowledged for their anti-inflammatory attributes, largely achieved by competing with ω-6 PUFA for enzymatic conversion [42]. Notably, ω-3 PUFA also serve as precursors for a distinct class of eicosanoids, including leukotriene B5, which possesses either negligible or anti-inflammatory properties, and anti-inflammatory lipid mediators known as pro-resolving mediators [43, 44].

The ω-3 PUFA, EPA and DHA, are known to exert their anti-inflammatory effects in the enterocytes through the partial replacement of AA in cellular phospholipids, the inhibition of several inflammatory signaling pathways, the activation of peroxisome proliferator-activated receptor (PPAR)-γ and G-protein coupled receptor, which subsequently inhibit the action of the proinflammatory transcription factor nuclear kappa-light-chain-enhancer of activated B cells (NF-κB) [45].

Given that both AA and ω-3 PUFA share the same enzymes, it has been observed that the incorporation of ω-3 PUFA into the cell membrane of erythrocyte occurs at the expense of AA [46] with ω-3 being preferentially utilized [41]. The data collected from this FCE study in cats and the previous study in CE dogs [23] allowed us to hypothesize that the inflammatory process, through upregulation of phospholipase A2 expression, may lead to a high rate of detachment from cell membranes of AA and a subsequent imbalance of substrate competition and the accumulation of ω-3 in membranes. These results seem in agreement with a recent study by Marsilio and coll. [47] in which an increase of fecal arachidonate was found in cats, suggesting mucosal upregulation during inflammation and subsequent leakage into the lumen. Similarly, dogs with FRE tended to have greater AA in feces when compared to the control group, suggesting excessive membrane destruction in sick dogs or to greater production of this fatty acid to repair cellular damage at the intestinal level [48].

The ω-6/ω-3 ratio, which is also referred to as the inflammatory risk index in humans [49], can be managed through dietary management to reduce intestinal inflammation in humans and animals, and the PUFA balance is a usefulness index for assessing the overall effects of PUFA on membranes [50]. This index could aid in determining the role of nutraceutical intervention and, specifically, the appropriateness of ω-3 supplementation in feline CE. Recently, researchers have investigated the ω-3 index in both dogs and cats, which reflects the content of EPA and DHA in erythrocytes expressed as a percentage of total RBC membrane FAs [32]. These indexes can aid in estimating the appropriate amount of ω-3 supplementation required in cats affected by CE.

Other than EPA and DHA, in this study, the ω-3 PUFA DPA was considered in the FA lipidomic profile of cats. DPA (22:5) is the precursor of the DHA, and it derives from the EPA (20:5) by the elongase enzymatic activity [51]. DHA levels in RBC membranes of dogs were negligible [23, 52]. On the other hand, adult cats can produce DPA in liver and plasma [28] representing approximately 0.5% of the total FA content of RBC membranes of HC in the present study, with EPA and DHA approximately 0.9% each, and interestingly DPA was significantly increased in FCE. Like EPA and DHA, DPA is a substrate for the synthesis of specialized pro-resolving mediators with biological activity (i.e., resolvins and protectins), suggesting a role in the resolution of inflammation [53, 54]. Moreover, the rapid conversion between EPA and DPA indicates the possibility that DPA can be a potential storage form for EPA [54], suggesting an impact of DPA on various aspects of chronic inflammation in cats.

Similarly to the previous findings in the study on dogs [23], the composition of RBC membranes in terms of FA percentages in cats was not capable of discriminating among the different forms of chronic enteropathy (FRE, IBD, LGITL). This outcome is not surprising, as currently there is no single diagnostic criterion or established biomarker available that can reliably differentiate IBD from LGITL of cats [4, 55–58]. Furthermore, it is noteworthy that both conditions often coexist within the same individual, which poses a challenge in distinguishing the various forms of FCE [5]. Another plausible explanation for this difficulty could be that the chronic inflammatory process characterizes all three forms independently of their specific triggering cause [5].

Hypocobalaminemia has been frequently reported in FCE and has been demonstrated to have a correlation with the severity of clinical signs in cats [59, 60]. Along with folate, cobalamin serves as an indicator of jejunal and ileal malabsorption, respectively, providing valuable information about the location of the disease [61]. In the present study hypocobalaminemic cats exhibited an increase in oleic acid, vaccenic acid, and total MUFA. Subsequently, elevated activity of Δ-9 desaturase was observed, which may account for the substantial accumulation of MUFA in these cases. These findings support the hypothesis that this enzyme may play a crucial role in the pathogenesis of FCE. Similarly, in dogs, a negative correlation was observed between cobalaminemia and vaccenic acid levels in RBC membranes, with an increase of vaccenic acid in dogs with CE that did not respond to therapy [23].

Among FCE cats, an increase in FCEAI was found to be associated with higher levels of LA, DPA, ω-6 PUFA, and total PUFA. This higher rate of PUFA incorporation impacts the distribution of FAs in RBC membranes, leading to lower levels of SFA at higher clinical activity index scores. We can also speculate that higher PUFA levels in RBC membranes may reflect a greater susceptibility to oxidative damage, which is known to occur during inflammation [62]. This finding supports the hypothesis that ω-3 may be upregulated in both mild and severe diseases, and their consumption may increase as the disease activity becomes more severe [63] as already suggested in dogs with CE [23].

While in CE dogs the FA-based RBC membrane lipidome did not appear to be influenced by the BCS [23], in FCE, lower values of BCS were associated with reduced levels of DHA and total ω-3 with subsequent significant increase of ω-6/ω-3. In these subjects, factors such as malabsorption, oxidative decomposition of PUFA, low desaturation activity, and rapid turnover of RBCs may all contribute to the lower DHA levels.

This study represents the initial examination of the RBC membrane FA profile in FCE. However, it is important to acknowledge several limitations. Firstly, the sample size was restricted, which may have reduced the statistical power of the analyses. Moreover, the absence of a standardized diet before enrolling the cats could potentially impact the interpretation of the results, and implementing a standardized, high-quality diet with known percentages of FA content could address this limitation. Finally, conducting follow-up sampling could have provided valuable insight into treatment response.

The study suggests that future investigations on the FA lipidomic profile of RBC membranes in FCE could offer potential benefits in monitoring treatment response, personalized nutraceutical approaches, and identifying markers of disease relapse. Prospective studies under controlled conditions and with standardized diets would be necessary to further explore the effects of diet and treatment on lipidomic changes. Recent advancements in lipidomics provide opportunities for comprehensive lipidome mapping and monitoring in humans and animals, including cats, to understand the impact of chronic enteropathy on animal health.

## Conclusion

In summary, the present study highlights significant FA changes in RBC membrane in FCE compared to HC with the most notable differences were observed in the ω-3 PUFA (DPA, DHA, total ω-3 PUFA). A previous study suggested that changes in RBC membranes, in particular ω-3 PUFA, seem to reflect changes in gastrointestinal tissue [64], suggesting that FA-based RBC membrane lipidome approach may represent a valid and less invasive test for investigating the health of the gastrointestinal tract.

The applicability FA-based RBC as an indicator for specific health outcomes, including feline chronic enteropathy, requires further investigation to be confirmed.

## Supporting information

S1 Table Median values with interquartile ranges in brackets of the single FAs, total FA contents of red blood cells membranes (total SFA, total MUFA, and total PUFA), homeostasis indexes (SFA/MUFA, ω-6/ω-3, UI, PI, and PUFA balance) and enzyme activity indexes (EI, Δ9DI, Δ6DI, Δ5DI) in the different groups of FCE cats: Food-responsive enteropathy (FRE), inflammatory bowel disease (IBD) and low-grade intestinal T-cell lymphoma (LGITL). SFA: Saturated Fatty Acids; MUFA: Monounsaturated Fatty Acids; PUFA: Polyunsaturated Fatty Acids; UI: Unsaturation index; PI: Peroxidation index.
(DOCX)

## References

[1] Jergens AE. Feline idiopathic inflammatory bowel disease: what we know and what remains to be unraveled. J Feline Med Surg. 2012 Jul;14(7):445–58. doi: 10.1177/1098612X12451548 22736679 PMC10822384

[2] Barrs VR, Beatty JA. Feline alimentary lymphoma: 1. Classification, risk factors, clinical signs and non-invasive diagnostics. J Feline Med Surg. 2012 Mar;14(3):182–90. doi: 10.1177/1098612X12439265 22370860 PMC10822432

[3] Barrs VR, Beatty JA. Feline alimentary lymphoma: 2. Further diagnostics, therapy and prognosis. J Feline Med Surg. 2012 Mar;14(3):191–201. doi: 10.1177/1098612X12439266 22370861 PMC10822435

[4] Marsilio S, Freiche V, Johnson E, Leo C, Langerak AW, Peters I, et al. ACVIM consensus statement guidelines on diagnosing and distinguishing low-grade neoplastic from inflammatory lymphocytic chronic enteropathies in cats. J Vet Intern Med. 2023;37(3):794–816. doi: 10.1111/jvim.16690 37130034 PMC10229359

[5] Marsilio S. Differentiating Inflammatory Bowel Disease from Alimentary Lymphoma in Cats: Does It Matter? Vet Clin North Am Small Anim Pract. 2021 Jan;51(1):93–109. doi: 10.1016/j.cvsm.2020.09.009 33187624

[6] Marsilio S. Feline chronic enteropathy. J Small Anim Pract. 2021 Jun;62(6):409–19. doi: 10.1111/jsap.13332 33821508

[7] Boldyreva LV, Morozova MV, Saydakova SS, Kozhevnikova EN. Fat of the Gut: Epithelial Phospholipids in Inflammatory Bowel Diseases. Int J Mol Sci. 2021 Oct 28;22(21):11682. doi: 10.3390/ijms222111682 34769112 PMC8584226

[8] Diab J, Hansen T, Goll R, Stenlund H, Ahnlund M, Jensen E, et al. Lipidomics in Ulcerative Colitis Reveal Alteration in Mucosal Lipid Composition Associated With the Disease State. Inflamm Bowel Dis. 2019 Oct 18;25(11):1780–7. doi: 10.1093/ibd/izz098 31077307

[9] Kwon J, Lee C, Heo S, Kim B, Hyun CK. DSS-induced colitis is associated with adipose tissue dysfunction and disrupted hepatic lipid metabolism leading to hepatosteatosis and dyslipidemia in mice. Sci Rep. 2021 Mar 5;11(1):5283. doi: 10.1038/s41598-021-84761-1 33674694 PMC7935975

[10] Nyström N, Prast-Nielsen S, Correia M, Globisch D, Engstrand L, Schuppe-Koistinen I, et al. Mucosal and Plasma Metabolomes in New-onset Paediatric Inflammatory Bowel Disease: Correlations with Disease Characteristics and Plasma Inflammation Protein Markers. J Crohns Colitis. 2023 Apr 3;17(3):418–32. doi: 10.1093/ecco-jcc/jjac149 36219554 PMC10069620

[11] Scoville EA, Allaman MM, Brown CT, Motley AK, Horst SN, Williams CS, et al. Alterations in Lipid, Amino Acid, and Energy Metabolism Distinguish Crohn’s Disease from Ulcerative Colitis and Control Subjects by Serum Metabolomic Profiling. Metabolomics. 2018 Jan;14(1):17. doi: 10.1007/s11306-017-1311-y 29681789 PMC5907923

[12] Ye J, Haskey N, Dadlani H, Zubaidi H, Barnett JA, Ghosh S, et al. Deletion of mucin 2 induces colitis with concomitant metabolic abnormalities in mice. Am J Physiol Gastrointest Liver Physiol. 2021 May 1;320(5):G791–803. doi: 10.1152/ajpgi.00277.2020 33728986

[13] Ma C, Vasu R, Zhang H. Vol. 2019, Mediators of Inflammation. Hindawi; 2019 [cited 2021 Jan 8]. p. e8495913 The Role of Long-Chain Fatty Acids in Inflammatory Bowel Disease. https://www.hindawi.com/journals/mi/2019/8495913/10.1155/2019/8495913 PMC6874876 31780872

[14] Ferreri C, Masi A, Sansone A, Giacometti G, Larocca AV, Menounou G, et al. Fatty Acids in Membranes as Homeostatic, Metabolic and Nutritional Biomarkers: Recent Advancements in Analytics and Diagnostics. Diagnostics (Basel). 2016 Dec 22;7(1). doi: 10.3390/diagnostics7010001 28025506 PMC5373010

[15] Marion-Letellier R, Savoye G, Beck PL, Panaccione R, Ghosh S. Polyunsaturated fatty acids in inflammatory bowel diseases: a reappraisal of effects and therapeutic approaches. Inflamm Bowel Dis. 2013 Mar;19(3):650–61. doi: 10.1097/MIB.0b013e3182810122 23328774

[16] Michalak A, Mosińska P, Fichna J. Polyunsaturated Fatty Acids and Their Derivatives: Therapeutic Value for Inflammatory, Functional Gastrointestinal Disorders, and Colorectal Cancer. Front Pharmacol. 2016;7:459. doi: 10.3389/fphar.2016.00459 27990120 PMC5131004

[17] Alhouayek M, Ameraoui H, Muccioli GG. Bioactive lipids in inflammatory bowel diseases—From pathophysiological alterations to therapeutic opportunities. Biochim Biophys Acta Mol Cell Biol Lipids. 2020 Nov 4;1866(2):158854. doi: 10.1016/j.bbalip.2020.158854 33157277

[18] Barnig C, Bezema T, Calder PC, Charloux A, Frossard N, Garssen J, et al. Activation of Resolution Pathways to Prevent and Fight Chronic Inflammation: Lessons From Asthma and Inflammatory Bowel Disease. Front Immunol. 2019;10:1699. doi: 10.3389/fimmu.2019.01699 31396220 PMC6664683

[19] Bühner S, Nagel E, Körber J, Vogelsang H, Linn T, Pichlmayr R. Ileal and colonic fatty acid profiles in patients with active Crohn’s disease. Gut. 1994 Oct;35(10):1424–8. doi: 10.1136/gut.35.10.1424 7959199 PMC1375018

[20] Fernández-Bañares F, Esteve-Comas M, Mañé J, Navarro E, Bertrán X, Cabré E, et al. Changes in mucosal fatty acid profile in inflammatorybowel disease and in experimental colitis: a common response to bowel inflammation. Clin Nutr. 1997 Aug;16(4):177–83. doi: 10.1016/s0261-5614(97)80003-9 16844596

[21] Longo S, Chieppa M, Cossa LG, Spinelli CC, Greco M, Maffia M, et al. New Insights into Inflammatory Bowel Diseases from Proteomic and Lipidomic Studies. Proteomes. 2020 Aug 10;8(3). doi: 10.3390/proteomes8030018 32784952 PMC7565982

[22] Ueda Y, Kawakami Y, Kunii D, Okada H, Azuma M, Le DSNT, et al. Elevated concentrations of linoleic acid in erythrocyte membrane phospholipids in patients with inflammatory bowel disease. Nutr Res. 2008 Apr;28(4):239–44. doi: 10.1016/j.nutres.2008.02.005 19083414

[23] Crisi PE, Luciani A, Di Tommaso M, Prasinou P, De Santis F, Chatgilialoglu C, et al. The Fatty Acid-Based Erythrocyte Membrane Lipidome in Dogs with Chronic Enteropathy. Animals (Basel). 2021 Sep 5;11(9):2604. doi: 10.3390/ani11092604 34573570 PMC8469057

[24] Sung CH, Pilla R, Marsilio S, Chow B, Zornow KA, Slovak JE, et al. Fecal Concentrations of Long-Chain Fatty Acids, Sterols, and Unconjugated Bile Acids in Cats with Chronic Enteropathy. Animals (Basel). 2023 Aug 30;13(17):2753. doi: 10.3390/ani13172753 37685017 PMC10486672

[25] Jergens A e., Crandell J m., Evans R, Ackermann M, Miles K g., Wang C. A Clinical Index for Disease Activity in Cats with Chronic Enteropathy. Journal of Veterinary Internal Medicine. 2010;24(5):1027–33. doi: 10.1111/j.1939-1676.2010.0549.x 20584141

[26] Kathrani A. Dietary and Nutritional Approaches to the Management of Chronic Enteropathy in Dogs and Cats. Vet Clin North Am Small Anim Pract. 2021 Jan;51(1):123–36. doi: 10.1016/j.cvsm.2020.09.005 33131914

[27] Prasinou P, Crisi PE, Chatgilialoglu C, Di Tommaso M, Sansone A, Gramenzi A, et al. The Erythrocyte Membrane Lipidome of Healthy Dogs: Creating a Benchmark of Fatty Acid Distribution and Interval Values. Front Vet Sci. 2020;7:502. doi: 10.3389/fvets.2020.00502 32974399 PMC7472600

[28] Bauer JJE. Essential fatty acid metabolism in dogs and cats. R Bras Zootec. 2008 Jul;37:20–7.

[29] FEDIAF | Nutritional Guidelines [Internet]. [cited 2024 May 5]. https://europeanpetfood.org/self-regulation/nutritional-guidelines/

[30] Bian Z, Jian X, Liu G, Jian S, Wen J, Zhang H, et al. Wet-food diet promotes the recovery from surgery of castration and control of body weight in adult young cats. J Anim Sci. 2023 Jan 3;101:skad039. doi: 10.1093/jas/skad039 36734030 PMC9997781

[31] Scaioli E, Liverani E, Belluzzi A. The Imbalance between n-6/n-3 Polyunsaturated Fatty Acids and Inflammatory Bowel Disease: A Comprehensive Review and Future Therapeutic Perspectives. Int J Mol Sci. 2017 Dec 5;18(12):2619. doi: 10.3390/ijms18122619 29206211 PMC5751222

[32] Harris WS, Jackson KH, Carlson H, Hoem N, Dominguez TE, Burri L. Derivation of the Omega-3 Index from EPA and DHA Analysis of Dried Blood Spots from Dogs and Cats. Vet Sci. 2022 Dec 26;10(1):13. doi: 10.3390/vetsci10010013 36669014 PMC9863621

[33] James C, Rodriguez-Zas SL, de Godoy MRC. PSXI-32 Effects of algae DHA on fatty acid profile of plasma red blood cell membrane and fecal microbiota of adult cats. J Anim Sci. 2020 Nov 30;98(Suppl 4):319.

[34] Sun Q, Ma J, Campos H, Hankinson SE, Hu FB. Comparison between plasma and erythrocyte fatty acid content as biomarkers of fatty acid intake in US women. Am J Clin Nutr. 2007 Jul;86(1):74–81. doi: 10.1093/ajcn/86.1.74 17616765

[35] Gunes O, Tascilar E, Sertoglu E, Tas A, Serdar MA, Kaya G, et al. Associations between erythrocyte membrane fatty acid compositions and insulin resistance in obese adolescents. Chem Phys Lipids. 2014 Dec;184:69–75. doi: 10.1016/j.chemphyslip.2014.09.006 25262585

[36] Jauregibeitia I, Portune K, Rica I, Tueros I, Velasco O, Grau G, et al. Fatty Acid Profile of Mature Red Blood Cell Membranes and Dietary Intake as a New Approach to Characterize Children with Overweight and Obesity. Nutrients. 2020 Nov 10;12(11):3446. doi: 10.3390/nu12113446 33182783 PMC7696547

[37] Sinclair AJ, McLean JG, Monger EA. Metabolism of linoleic acid in the cat. Lipids. 1979 Nov;14(11):932–6. doi: 10.1007/BF02533508 513981

[38] Rivers JPW, Sinclair AJ, Crawford MA. Inability of the cat to desaturate essential fatty acids. Nature. 1975 Nov;258(5531):171–3. doi: 10.1038/258171a0 1186900

[39] Pawlosky R, Barnes A, Salem N. Essential fatty acid metabolism in the feline: relationship between liver and brain production of long-chain polyunsaturated fatty acids. J Lipid Res. 1994 Nov;35(11):2032–40. 7868981

[40] Ferreri C, Chatgilialoglu C. Role of fatty acid-based functional lipidomics in the development of molecular diagnostic tools. Expert Rev Mol Diagn. 2012 Sep;12(7):767–80. doi: 10.1586/erm.12.73 23153242

[41] Schmitz G, Ecker J. The opposing effects of n-3 and n-6 fatty acids. Prog Lipid Res. 2008 Mar;47(2):147–55. doi: 10.1016/j.plipres.2007.12.004 18198131

[42] Calder PC. Omega-3 Fatty Acids and Inflammatory Processes. Nutrients. 2010 Mar 18;2(3):355–74. doi: 10.3390/nu2030355 22254027 PMC3257651

[43] Liu C, Fan D, Lei Q, Lu A, He X. Roles of Resolvins in Chronic Inflammatory Response. International Journal of Molecular Sciences. 2022 Jan;23(23):14883. doi: 10.3390/ijms232314883 36499209 PMC9738788

[44] Serhan CN, Levy BD. Resolvins in inflammation: emergence of the pro-resolving superfamily of mediators. J Clin Invest. 2018 Jul 2;128(7):2657–69. doi: 10.1172/JCI97943 29757195 PMC6025982

[45] Durkin LA, Childs CE, Calder PC. Omega-3 Polyunsaturated Fatty Acids and the Intestinal Epithelium-A Review. Foods. 2021 Jan 19;10(1):199. doi: 10.3390/foods10010199 33478161 PMC7835870

[46] Verbrugghe A, Janssens GPJ, Van de Velde H, Cox E, De Smet S, Vlaeminck B, et al. Failure of a dietary model to affect markers of inflammation in domestic cats. BMC Vet Res. 2014 May 4;10:104. doi: 10.1186/1746-6148-10-104 24885092 PMC4016662

[47] Marsilio S, Chow B, Hill SL, Ackermann MR, Estep JS, Sarawichitr B, et al. Untargeted metabolomic analysis in cats with naturally occurring inflammatory bowel disease and alimentary small cell lymphoma. Sci Rep. 2021 Apr 28;11(1):9198. doi: 10.1038/s41598-021-88707-5 33911166 PMC8080598

[48] Higueras C, Rey AI, Escudero R, Díaz-Regañón D, Rodríguez-Franco F, García-Sancho M, et al. Short-Chain and Total Fatty Acid Profile of Faeces or Plasma as Predictors of Food-Responsive Enteropathy in Dogs: A Preliminary Study. Animals (Basel). 2021 Dec 31;12(1):89. doi: 10.3390/ani12010089 35011195 PMC8749849

[49] Amézaga J, Arranz S, Urruticoechea A, Ugartemendia G, Larraioz A, Louka M, et al. Altered Red Blood Cell Membrane Fatty Acid Profile in Cancer Patients. Nutrients. 2018 Dec 1;10(12). doi: 10.3390/nu10121853 30513730 PMC6315925

[50] Abbott SK, Else PL, Atkins TA, Hulbert AJ. Fatty acid composition of membrane bilayers: importance of diet polyunsaturated fat balance. Biochim Biophys Acta. 2012 May;1818(5):1309–17. doi: 10.1016/j.bbamem.2012.01.011 22285120

[51] Richter CK, Bisselou KS, Nordgren T, Smith L, Appiah AK, Hein N, et al. n-3 docosapentaenoic acid (DPA) intake and relationship to plasma long-chain n-3 fatty acid concentrations in the United States: NHANES 2003–2014. Lipids. 2019 Apr;54(4):221–30. 31025717 10.1002/lipd.12146 PMC6681819

[52] Ghasemi Fard S, Cameron-Smith D, Sinclair AJ. n-3 Docosapentaenoic acid: the iceberg n-3 fatty acid. Curr Opin Clin Nutr Metab Care. 2021 Mar 1;24(2):134–8. 33315722 10.1097/MCO.0000000000000722

[53] Julliard WA, Myo YPA, Perelas A, Jackson PD, Thatcher TH, Sime PJ. Specialized pro-resolving mediators as modulators of immune responses. Semin Immunol. 2022 Jan;59:101605. doi: 10.1016/j.smim.2022.101605 35660338 PMC9962762

[54] Skulas-Ray AC, Flock MR, Richter CK, Harris WS, West SG, Kris-Etherton PM. Red Blood Cell Docosapentaenoic Acid (DPA n-3) is Inversely Associated with Triglycerides and C-reactive Protein (CRP) in Healthy Adults and Dose-Dependently Increases Following n-3 Fatty Acid Supplementation. Nutrients. 2015 Aug 4;7(8):6390–404. doi: 10.3390/nu7085291 26247967 PMC4555130

[55] Karra DA, Chadwick CC, Stavroulaki EM, Pitropaki MN, Flouraki E, Allenspach K, et al. Fecal acute phase proteins in cats with chronic enteropathies. J Vet Intern Med. 2023;37(5):1750–9. doi: 10.1111/jvim.16802 37401847 PMC10473003

[56] Love EK, Leibman NF, Ringold R, Lamb K. Serum haptoglobin concentrations in feline inflammatory bowel disease and small-cell alimentary lymphoma: a potential biomarker for feline chronic enteropathies. J Feline Med Surg. 2021 Oct;23(10):959–64. doi: 10.1177/1098612X21991448 33541236 PMC11197119

[57] Riggers DS, Xenoulis PG, Karra DA, Enderle LL, Köller G, Böttcher D, et al. Fecal Calprotectin Concentrations in Cats with Chronic Enteropathies. Vet Sci. 2023 Jun 28;10(7):419. doi: 10.3390/vetsci10070419 37505825 PMC10385529

[58] Zornow KA, Slovak JE, Lidbury JA, Suchodolski JS, Steiner JM. Fecal S100A12 concentrations in cats with chronic enteropathies. J Feline Med Surg. 2023 Mar;25(3):1098612X231164273. doi: 10.1177/1098612X231164273 36995216 PMC10812014

[59] Maunder CL, Day MJ, Hibbert A, Steiner JM, Suchodolski JS, Hall EJ. Serum cobalamin concentrations in cats with gastrointestinal signs: correlation with histopathological findings and duration of clinical signs. J Feline Med Surg. 2012 Oct;14(10):686–93. doi: 10.1177/1098612X12447730 22577047 PMC11104096

[60] Ruaux CG, Steiner JM, Williams DA. Early biochemical and clinical responses to cobalamin supplementation in cats with signs of gastrointestinal disease and severe hypocobalaminemia. J Vet Intern Med. 2005;19(2):155–60. doi: 10.1892/0891-6640(2005)19&lt;155:ebacrt&gt;2.0.co;2 15822558

[61] Dossin O. Laboratory tests for diagnosis of gastrointestinal and pancreatic diseases. Top Companion Anim Med. 2011 May;26(2):86–97. doi: 10.1053/j.tcam.2011.02.005 21596348 PMC7104967

[62] Ferreri C, Grabovskiy SA, Aoun M, Melchiorre M, Kabal’nova N, Feillet-Coudray C, et al. Trans fatty acids: chemical synthesis of eicosapentaenoic acid isomers and detection in rats fed a deodorized fish oil diet. Chem Res Toxicol. 2012 Mar 19;25(3):687–94. doi: 10.1021/tx200467c 22283477

[63] Wiese DM, Horst SN, Brown CT, Allaman MM, Hodges ME, Slaughter JC, et al. Serum Fatty Acids Are Correlated with Inflammatory Cytokines in Ulcerative Colitis. PLoS One [Internet]. 2016 May 26 [cited 2020 Apr 4];11(5). Available from: https://www.ncbi.nlm.nih.gov/pmc/articles/PMC4882051/ doi: 10.1371/journal.pone.0156387 27227540 PMC4882051

[64] Gurzell EA, Wiesinger J, Morkam C, Hemmrich S, Harris WS, Fenton JI. Is the omega-3 index a valid marker of intestinal membrane phospholipid EPA+DHA content? Prostaglandins Leukot Essent Fatty Acids. 2014 Sep;91(3):87–96. doi: 10.1016/j.plefa.2014.04.001 24913088 PMC4127132

