# Supplementary material for: Evaluation of the fatty acid-based erythrocyte membrane lipidome in cats with food responsive enteropathy, inflammatory bowel disease and low-grade intestinal T-cell lymphoma
Source: PLoS One. 2024 Jul 29;19(7):e0307757. doi: 10.1371/journal.pone.0307757 (PMC11285949; doi:10.1371/journal.pone.0307757)
Supplement: S1 Table — SFA: Saturated Fatty Acids; MUFA: Monounsaturated Fatty Acids; PUFA: Polyunsaturated Fatty Acids; UI: Unsaturation index; PI: Peroxidation index. (DOCX) [file pone.0307757.s001.docx]

**Supporting information (Tables)**

**S1 Table. Median values with interquartile ranges in brackets of the single FAs, total FA contents of red blood cells membranes (total SFA, total MUFA, and total PUFA), homeostasis indexes (SFA/MUFA, ω-6/ω-3, UI, PI, and PUFA balance) and enzyme activity indexes (EI, Δ9DI, Δ6DI, Δ5DI) in the different groups of FCE cats: food-responsive enteropathy (FRE), inflammatory bowel disease (IBD) and low-grade intestinal T-cell lymphoma (LGITL).**

| **Variable** | **FRE (n=17)**  **Median (IQR)** | **IBD (n=15)**  **Median (IQR)** | **LGITL (n=9)**  **Median (IQR)** | **p value** |
| --- | --- | --- | --- | --- |
| C16:0 **-** Palmitic Acid | 19.32 (17.8-24.8) | 20.04 (17.4-22.5) | 17.68 (16.5-19.9) | 0.6029 |
| C16:1 **-** Palmitoleic Acid | 0.13 (0.10-0.26) | 0.17 (0.08-0.20) | 0.15 (0.12-0.20) | 0.9748 |
| C18:0 **-** Stearic Acid | 22.3 (20.7-24.0) | 22.5 (20.7-25.2) | 23.1 (20.1-24.1) | 0.7802 |
| 9c,C18:1 **-** Oleic Acid | 10.8 (7.97-12.3) | 9.42 (8.07-12.1) | 9.43 (8.07-11.2) | 0.9178 |
| 11c,C18:1 **-** Vaccenic Acid | 1.77 (1.28-2.21) | 1.90 (1.60-2.44) | 1.82 (1.71-2.21) | 0.4407 |
| LA ω-6 **-** C18:2 **-** Linoleic Acid | 20.0 (15.84-23.31) | 21.8 (18.9-23.8) | 19.8 (18.5-23.5) | 0.4200 |
| DGLA ω-6 **-** C20:3 Dihomogammalinolenic Acid | 0.70 (0.53-1.03) | 0.76 (0.64-1.07) | 0.96 (0.66-1.22) | 0.5165 |
| ARA ω-6 **-** C20:4 **-** Arachidonic Acid | 19.9 (14.5-24.4) | 19.6 (14.7-21.3) | 20.4 (16.7- 26.4) | 0.3896 |
| EPA ω-3 **-** C20:5 **-** Eicosapentaenoic Acid | 1.44 (0.72-2.60) | 1.36 (0.56-2.48) | 1.90 (0.79-2.99) | 0.8176 |
| DPA ω-3 **-** C22:5 **-** Docosapentaenoic Acid | 0.62 (0.53-0.84) | 0.74 (0.43-0.88) | 0.80 (0.64-0.98) | 0.3209 |
| DHA ω-3 **-** C22:6 **-** Docosahexaenoic Acid | 1.30 (0.33-2.11) | 1.36 (0.68-1.58) | 1.42 (0.99-2.78) | 0.8176 |
| Total SFA | 41.6 (39.4-46.4) | 43.3 (40.0-45.5) | 41.1 (38.4-44.3) | 0.6912 |
| Total MUFA | 12.9 (9.42-14.6) | 11.4 (10.1-15.1) | 11.9 (9.97-13.8) | 0.8935 |
| Total PUFA | 45.9 (39.7-49.5) | 43.5 (41.6-47.6) | 46.1 (43.3-51.1) | 0.6523 |
| ω-6 PUFA | 41.9 (34.6-45.0) | 40.4 (35.6-44.2) | 41.2 (39-47.4) | 0.7660 |
| ω-3 PUFA | 3.27 (1.86-5.81) | 3.09 (2.52-4.77) | 3.93 (2.97-5.85) | 0.5621 |
| ω-6/ω-3 ratio | 14.5 (6.95-21.0) | 13.0 (8.33-16.3) | 10.6 (7.01- 15.1) | 0.6005 |
| SFA/MUFA | 3.64 (2.80-4.33) | 3.60 (2.84-4.19) | 3.81 (2.91-4.06) | 0.9092 |
| PUFA balance | 6.43 (4.54-12.9) | 7.12 (5.75-10.7) | 8.56 (6.25-12.5) | 0.6005 |
| UI | 156.6 (138.4-167.1) | 152.4 (136.9-162.5) | 171.1 (154.1-173) | 0.1423 |
| PI | 136.9 (109.4-147.7) | 126.1 (97.4-142.2) | 143(123.8-149.6) | 0.2847 |
| Elongase-6 activity | 1.16 (0.92-1.25) | 1.16 (1.01-1.29) | 1.23 (1.09-1.40) | 0.4579 |
| Delta-9 desaturase | 0.44 (0.33-0.57) | 0.41 (0.35-0.48) | 0.42 (0.35-0.50) | 0.9527 |
| Delta-6 desaturase | 0.03 (0.02-0.06) | 0.03 (0.02-0.04) | 0.04 (0.02-0.06) | 0.8493 |
| Delta-5 desaturase | 27.3 (18.6-31.2) | 23 (15.3-30.75) | 23.0 (14.0-30.5) | 0.6029 |

IQR: interquartile range; SFA: Saturated Fatty Acids; MUFA: Monounsaturated Fatty Acids; PUFA: Polyunsaturated Fatty Acids; UI: Unsaturation index; PI: Peroxidation index.
